# Development of Sarcoidosis Subsequent to Temporary Corticosteroid Treatment in a Patient With Rheumatoid Arthritis

**DOI:** 10.1002/rcr2.70364

**Published:** 2025-10-02

**Authors:** Tatsuhiro Itami, Motoyasu Kato, Yuki Nakashima, Shunichi Kataoka, Haruki Hirakawa, Ryoko Murashima, Eri Hayashi, Nagisa Yoshihara, Takuo Hayashi, Rei Watanabe, Naoto Tamura, Kazuhisa Takahashi

**Affiliations:** ^1^ Department of Respiratory Medicine Juntendo University Graduate School of Medicine Bunkyo‐ku Tokyo Japan; ^2^ Department of Internal Medicine and Rheumatology Juntendo University Graduate School of Medicine Bunkyo‐ku Tokyo Japan; ^3^ Department of Dermatology and Allergology Juntendo University Graduate School of Medicine Bunkyo‐ku Tokyo Japan; ^4^ Department of Human Pathology Juntendo University Graduate School of Medicine Bunkyo‐ku Tokyo Japan

**Keywords:** corticosteroid, immune reconstitution inflammatory syndrome, rheumatoid arthritis, sarcoidosis

## Abstract

A 69‐year‐old Japanese woman was diagnosed with rheumatoid arthritis and treated with tacrolimus because of worsening symptoms of arthritis. Four months after tacrolimus initiation, corticosteroids were administered. Arthritis improved after 4 months of corticosteroid administration, and the corticosteroid treatment was terminated. However, 2 months after corticosteroid treatment cessation, the patient developed mediastinal and hilar lymph node swelling, subcutaneous node swelling, uveitis, and elevated serum sarcoidosis‐associated markers. Consequently, the patient was diagnosed with sarcoidosis, characterised by non‐serodermal epithelial granuloma cells with positive staining for anti‐ *Propionibacterium acnes* antibody. Notably, all symptoms improved without systemic steroid administration. Corticosteroids and tacrolimus can affect CD4+ lymphocyte function and number; in the present case, given the use of temporary corticosteroids, they may have caused immune reconstitution inflammatory syndrome‐like pathogenesis, which can result in transient sarcoidosis.

## Graphical abstract

Two months after corticosteroid treatment for RA, the patient exhibited mediastinal and hilar lymph node swelling, subcutaneous node swelling, uveitis and was diagnosed with temporary sarcoidosis.

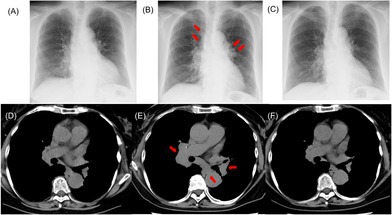

## 1 Introduction

Sarcoidosis is a disease of unknown aetiology in which inflammation occurs in various organs, especially the lungs, heart, eyes, skin, nerves, and lymph nodes resulting in the formation of non‐desmoplastic necrotic epithelioid cell granulomas.

Previous studies have documented the development of sarcoidosis and sarcoidosis‐like reactions that biological agents, such as anti‐TNFα inhibitors and immune checkpoint inhibitors, may have triggered through immune activation. Despite the implication of steroids and immunosuppressive drugs in sarcoidosis‐like reactions, no cases have been reported to date. In this study, we present the case of sarcoidosis that developed after temporary steroid treatment during tacrolimus administration for rheumatoid arthritis that subsequently resolved without intervention.

## 2 Case Report

**FIGURE 1:**
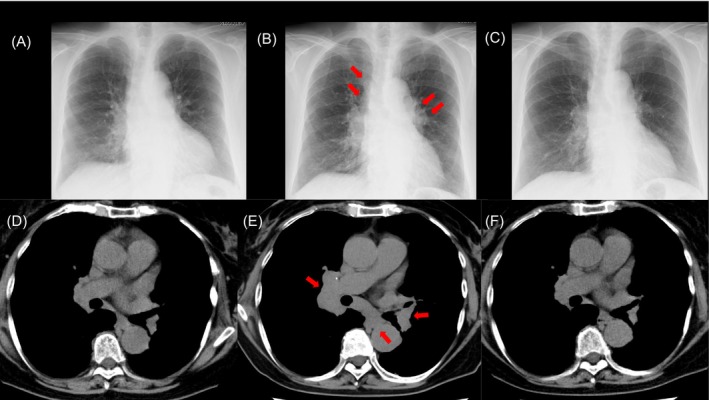
Changes in radiological findings. Chest radiograph findings (A) 4 months before sarcoidosis diagnosis, (B) at sarcoidosis diagnosis and (C) 4 months after sarcoidosis diagnosis. Chest high‐resolution computed tomography findings (D) 6 months before sarcoidosis diagnosis, (E) at sarcoidosis diagnosis, and (F) 6 months after sarcoidosis diagnosis.

**FIGURE 2:**
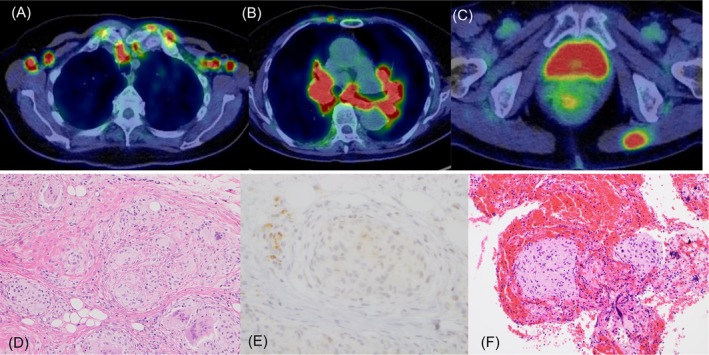
Positron emission tomography‐computed tomography and pathological findings. (A–D) Positron emission tomography‐computed tomography findings. (E) Haematoxylin and eosin stain of the subcutaneous nodule, (F) immunohistochemical stain of the subcutaneous nodule and (G) haematoxylin and eosin stain of the mediastinum lymph node.

A 69‐year‐old Japanese woman was diagnosed with rheumatoid arthritis (RA) with associated interstitial lung disease. Tacrolimus was initiated 1 year after the RA diagnosis. Given the absence of improvement in joint pain and swelling, prednisolone (5 mg/d) was initiated after 2 months of tacrolimus treatment. Following corticosteroid initiation, her symptoms improved and the steroid dosage was decreased by 2.5 mg each 2 months, and treatment was terminated 4 months after the corticosteroid initiation. Two months after corticosteroid therapy termination, chest radiography revealed bilateral enlarged hilar lymph nodes (Figure 1: at diagnosis, B; 4 months prior, A). Chest high‐resolution computed tomography revealed mediastinal, axial, and hilar lymph node swelling (Figure 1E; 1 year prior, Figure 1D). Furthermore, the patient exhibited foggy vision, and multiple irregular subcutaneous nodules were observed on both upper arms. Serum angiotensin‐converting enzyme (ACE, 39.9 U/L) and soluble interleukin‐2 receptor (sIL‐2R, 2320 U/mL) levels were elevated. Positron emission tomography‐computed tomography revealed fluorodeoxyglucose uptake in bilateral hilar and mediastinal lymphadenopathy, intramuscular accumulation in the left gluteus maximus, and abnormal accumulation in the sternocostal joints (SUV max 19.8; Figure 2A–D). An ophthalmologist diagnosed the patient with uveitis. Pathological examination revealed the presence of non‐serodermal epithelial granulomas (Figure 2E) that exhibited positive staining for anti‐ *Propionibacterium acnes* antibody [1] (Figure 2F). A transbronchial needle aspiration cytology specimen from a mediastinal lymph node revealed epithelioid granulomas (Figure 2G). Neither *Propionibacterium acnes* nor polymerase chain reaction evidence of tuberculosis or nontuberculous mycobacteria was identified in this specimen. Based on diagnostic criteria, the patient was diagnosed with sarcoidosis. Subsequently, following local treatment with a corticosteroid ointment, the uveitis improved. Finally, resolution occurred in lymph node swelling, subcutaneous nodes, and serum markers including serum angiotensin‐converting enzyme and soluble interleukin‐2 receptor (Figure 1C,F).

## 3 Discussion

Sarcoidosis has been suggested to be associated with autoimmune diseases such as RA [2]. This association is considered to result from involvement of immune responses in disease development. In addition, cases have been reported in which sarcoidosis relapsed or worsened following tapering or discontinuation of corticosteroid therapy [3]. Therefore, in the present case, although the patient had not been previously diagnosed with sarcoidosis, it is conceivable that a latent predisposition to sarcoidosis was present, which subsequently became manifest following corticosteroid tapering for RA.

Sarcoidosis occurs concurrently with increased lymphocyte numbers after highly active antiretroviral therapy for acquired immunodeficiency syndrome (AIDS), which represents a form of immune reconstitution inflammatory syndrome (IRIS). In most cases, the condition developed several months after the initiation of highly active antiretroviral therapy, at a time when CD4‐positive lymphocyte counts had increased and serum Human Immunodeficiency Virus (HIV)‐ribonucleic acid levels had decreased. CD4+ T lymphocytes accumulate at granulomatous inflammatory sites in patients with sarcoidosis, suggesting a potential role of CD4+ T lymphocytes in the pathogenesis of this condition. This evidence suggests increased susceptibility to sarcoidosis in patients with AIDS who undergo treatment and experience subsequent increases in CD4+ T lymphocyte counts.

Although IRIS commonly occurs during treatment in patients with AIDS, recent studies have reported IRIS in non‐HIV cases. In our patient with RA, temporary corticosteroid administration coincided with tacrolimus administration. Corticosteroids generally decrease CD4+ lymphocyte counts, while tacrolimus suppresses helper T‐cell function. Temporary corticosteroid administration is more likely to trigger sarcoidosis onset following compromised T‐cell function due to tacrolimus use. Although we did not measure the CD4+ lymphocyte count in the present case, given that the aetiology of sarcoidosis involves excessive immunity to the indigenous skin bacteria *Propionibacterium acnes* [1], and that IRIS results from excessive immunity to various pathogens, this hypothesis appears valid. Then, the concomitant use of tacrolimus, in addition to corticosteroids, may have contributed to the transient development of sarcoidosis in this case.

In patients diagnosed with sarcoidosis, increased levels of inflammatory cytokines, including tumour necrosis‐alpha and IL‐6, have been observed in both alveolar macrophages and bronchoalveolar lavage fluid [4]. The administration of tumour necrosis‐alpha inhibitors has been shown to markedly reduce granulomas in murine models of granulomatous disease; however, granulomatous lesions recur following treatment discontinuation [5]. It has been posited that steroids exert their effects on macrophages, thereby impeding the release of cytokines such as tumour necrosis factor‐alpha and interleukins 1, 2 and 6. The potential impact of steroids on these cytokines may have contributed to the manifestation of sarcoidosis in this particular instance.

In conclusion, the adjustment of immunosuppressive medications resulting in immune reconstitution could have lead to the development of sarcoidosis in a patient with RA.

## Author Contributions

T.I., M.K., Y.N., S.K., H.H., R.M., and E.H. followed the patient and managed the patient's treatment for RA, sarcoidosis, and interstitial lung disease. T.I., Y.N., and R.M. were responsible for bronchoscopy. T.H. evaluated lung pathological findings. N.Y. and R.W. evaluated skin pathological findings. T.I. and M.K. wrote the manuscript. M.K., R.W., N.T., and K.T. supervised the patient's management and this report. All authors read the manuscript and agreed the manuscript's publication. K.T. is an Editorial Board member of Respirology Case Reports and a co‐author of this article. He was excluded from all editorial decision‐making related to the acceptance of this article for publication.

## Ethics Statement

The report was approved by the Research Ethics Committee, Faculty of Medicine, Juntendo University (JHS24‐024).

## Consent

The authors declare that written informed consent was obtained for the publication of this manuscript and accompanying images using the consent form provided by the Journal.

## Conflicts of Interest

The authors declare no conflicts of interest.

## Data Availability

The data that support the findings of this study are available on request from the corresponding author. The data are not publicly available due to privacy or ethical restrictions.
